# Environmentally-Friendly High-Density Polyethylene-Bonded Plywood Panels

**DOI:** 10.3390/polym11071166

**Published:** 2019-07-08

**Authors:** Pavlo Bekhta, Ján Sedliačik

**Affiliations:** 1 Department of Wood-Based Composites, Cellulose, and Paper, Ukrainian National Forestry University, 79057 Lviv, Ukraine; 2 Department of Furniture and Wood Products, Technical University in Zvolen, 960 53 Zvolen, Slovakia

**Keywords:** alder plywood, high-density polyethylene film, bending strength, modulus of elasticity in bending, shear strength, thickness swelling, water absorption

## Abstract

Thermoplastic films exhibit good potential to be used as adhesives for the production of veneer-based composites. This work presents the first effort to develop and evaluate composites based on alder veneers and high-density polyethylene (HDPE) film. The effects of hot-pressing temperature (140, 160, and 180 °C), hot-pressing pressure (0.8, 1.2, and 1.6 MPa), hot-pressing time (1, 2, 3, and 5 min), and type of adhesives on the physical and mechanical properties of alder plywood panels were investigated. The effects of these variables on the core-layer temperature during the hot pressing of multiplywood panels using various adhesives were also studied. Three types of adhesives were used: urea–formaldehyde (UF), phenol–formaldehyde (PF), and HDPE film. UF and PF adhesives were used for the comparison. The findings of this work indicate that formaldehyde-free HDPE film adhesive gave values of mechanical properties of alder plywood panels that are comparable to those obtained with traditional UF and PF adhesives, even though the adhesive dosage and pressing pressure were lower than when UF and PF adhesives were used. The obtained bonding strength values of HDPE-bonded alder plywood panels ranged from 0.74 to 2.38 MPa and met the European Standard EN 314-2 for Class 1 plywood. The optimum conditions for the bonding of HDPE plywood were 160 °C, 0.8 MPa, and 3 min.

## 1. Introduction

The wood-based composites sector plays an important role in national economies in many countries. Plywood is widely used for different applications, such as construction, furniture manufacturing, means of transportation, packaging, decorative purposes, and many others. In comparison with conventional solid wood products, plywood has various advantages: increased dimensional stability, uniformity and higher mechanical strength, reduced processing cost, availability in larger sizes, better appearance, and biological benefits. On the other hand, one of the main disadvantages of plywood products is using a large amount of adhesive during its manufacture, which can be up to 20% of its total mass [1]. This disadvantage decreases the plywood product’s ecological balance and makes it less favorable than solid wood, especially when considering resins derived from petrochemical resources. Global production of plywood reached 157 mln m^3^ in 2017 [2]. To produce such an amount of plywood, approximately 15 mln tons of resin are used. Synthetic thermosetting resins based on phenol, urea, formaldehyde, and isocyanates are usually used.

Urea–formaldehyde (UF) resins are incombustible, provide good bonding strength, resistance to fluctuations of temperature, light, and corrosion, have a small curing time, simple manufacturing technology, and low production costs. However, they also have significant disadvantages, such as fragility, low water resistance, and significant emissions of formaldehyde. Phenol–formaldehyde (PF) resins can improve the bonding strength and water resistance, but they require a longer curing time, higher curing temperatures, higher production costs, and also emit phenol and formaldehyde [3]. The formaldehyde can irritate the eyes, respiratory and nervous systems, and possibly lead to cancer and leukemia. Therefore, formaldehyde was reclassified in 2004 by the International Agency for Research on Cancer (IARC) as “carcinogenic to humans (Group 1)” [4], compelling companies to reduce formaldehyde emission to lower levels.

Significant efforts have been made to reduce formaldehyde emissions from wood-based panels by the addition of various additives to the thermosetting resins or by the protection of the product with veneer, varnish, paint, and other coatings [5]. One of the possible directions is the creation of wood composites based on environmentally-friendly products, where thermoplastics (polyethylene, polypropylene, poly(vinyl chloride), and their copolymers) are used as adhesives. Already, there is a positive experience in the creation and use of wood composites based on thermoplastics [6,7,8,9,10,11,12,13,14,15,16,17,18,19]. Waste polyethylene can be used in the manufacture of oriented strand board (OSB) panels, resulting in the enhancement of thickness swelling, humidity, dimensional stability, water absorption, and screw withdrawal resistance [6]. Laminated veneer lumber (LVL) was manufactured using high-density polyethylene (HDPE) as a binding agent [7]. The properties of the composite boards were quite similar to or even better than those found in LVL made using thermosetting resin. The thermoplastic polymers were successfully used for coating of birch plywood [8,9,10]. Formaldehyde-free wood–plastic plywood has been successfully produced using thermoplastic polymers as wood adhesive [11,12,13,14,15,16,17,18,19].

The various thermoplastic polymers such as HDPE [7,12,13,14,15,17,19,20,21], polystyrene [16,22], polypropylene [18,21], or poly(vinyl chloride) [23,24] in different forms, such as textile fiber waste (polyurethane, polyamide-6) [21], recycled plastic shopping bags [7,11], or film [12,13,14,15,17,18,19,20,23,24,25] were used for veneer bonding.

The use of thermoplastic film as an adhesive for the bonding of veneer, apart from the fact that the plastic film is formaldehyde-free, has several other advantages compared with using liquid adhesives. Dry adhesive film is simpler to apply than wet adhesives; all of the untidy and unpleasant mixing and spreading operations in wet gluing are wholly removed from the plywood factory by the use of dry adhesive film. The dry adhesive film contains in each square meter of surface precisely the same quantity of adhesive, equal quality, uniform composition, exactly the same bond strength, and the same standard thickness [26].

Unfortunately, thermoplastic polymers are often hydrophobic, which leads to severe problems in the adhesion, causing poor mechanical properties [27]. Therefore, it is very important to promote the adhesion between a hydrophilic wood and a hydrophobic thermoplastic polymer, which can be done by using coupling agents [28,29], various surface treatments [23,24,30], thermal, or chemical modification of wood [25,27,31]. Some researchers used modified HDPE or polypropylene to manufacture plywood [32,33,34]. However, most of these approaches result in an obvious increase in cost and complexity of the preparation process. An alternative way of enhancing the physical and mechanical properties of plastic-bonded plywood is the modification of fabrication conditions, having an obvious advantage of low cost and easy processing [19].

Most of the mentioned studies used poplar, rarely eucalyptus or oak for bonding with thermoplastic polymers. No literature is available on using alder wood veneer. Alder is one of the most promising under-utilized wood species in Europe. Due to its workability and properties, alder can be considered as a suitable material for plywood manufacturing. Currently, producers of plywood in Ukraine often replace the traditional birch raw material with the alder raw material. This work presents the first effort to develop and evaluate composites based on alder veneers and HDPE film.

## 2. Materials and Methods

### 2.1. Materials

Rotary-cut alder wood veneer (*Alnus glutinosa* Goertn.) with dimensions of 300 mm × 300 mm × 1.6 mm and an average moisture content of 6% was used to make plywood panels. To minimize the influence of wood structure defects on the results of the experiment, the veneer sheets were selected and evaluated for the production of plywood panels. The veneer sheets were visually checked, and sheets without shocks, cracks, curling, and colors of more or less uniform thickness were selected. Observation on the wood appearance did not show any visible defects.

HDPE film with a thickness of 0.14 mm, density of 0.93 g/cm^3^ and melting point of 135 °C was used for the bonding of plywood samples. The plastic film was cut into the same dimensions as the veneers. UF and PF resins were also used for the comparison. UF and PF adhesives were prepared according to the manufacturer’s instructions. For the preparation of UF adhesive, 5% hardener (ammonium nitrate) and 15% filler (wheat flour) were used.

### 2.2. Manufacturing of Plywood Samples

**Figure 1:**
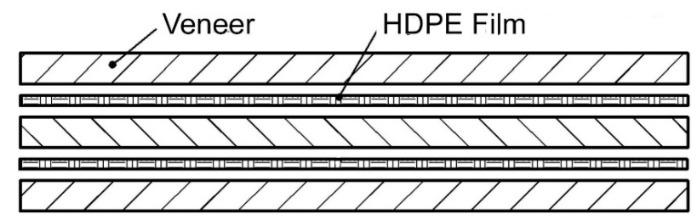
Structure of three-layer high-density polyethylene HDPE-bonded plywood panels.

Three-layer plywood samples were prepared. Instead of traditional UF and PF adhesives, a HDPE film was used as an adhesive for manufacturing the plywood samples. One sheet of HDPE film was incorporated between two veneer sheets, which were laid with the directions of the fiber perpendicular to each other. The laying of the dry HDPE film was very simple, and the design of the package is shown in Figure 1.

The influence of hot-pressing pressure (0.8, 1.2, and 1.6 MPa), hot-pressing temperature (140, 160, and 180 °C), and hot-pressing time (1, 2, 3, and 5 min) on the properties of plywood were evaluated. The pressing temperature depends on the adhesive. The melting temperature of the HDPE film was 135 °C, implying the value of the lower limit of hot-pressing temperature. This temperature must be over 135 °C, thus making HDPE flow and penetrate the alder veneers. By contrast, conventional plywood made from the commercial UF and PF resins is generally hot-pressed at approximately 105 and 145 °C, respectively. Therefore, a range of 140 to 180 °C was considered for hot pressing plywood panels using HDPE film as adhesive. The pressing conditions for manufacturing of plywood samples are given in Table 1. The physical and mechanical properties of HDPE-bonded plywood were compared with UF and PF plywood, and relevant plywood standards.

For the comparison, the plywood samples using UF and PF adhesives were produced according to the regimens usually used in practice. UF plywood samples were manufactured at the hot-pressing conditions: pressure of 1.8 MPa, temperature of 105 °C, time of 3 min, and an adhesive dosage of 160 g/m^2^. PF plywood samples were produced at a pressure of 1.8 MPa, temperature of 145 °C, time of 3 min, and adhesive dosage of 160 g/m^2^. The adhesive mixture was applied to the surface of the veneer by hand using a roller, and the open assembly time was about 5 min. The dosage of HDPE film at a thickness of 0.14 mm equals 130 g/m^2^, which was 19% less than in the case of using UF and PF adhesive.

After hot pressing, the plywood samples were subjected to a cold-press stage that was performed at room temperature for 5 min, which was used to reduce the distortion and stress of the plywood. Three replicate panels were manufactured for all the conditions and control.

After bonding, the plywood panels were air conditioned for 5 days. After air conditioning in a standard climate (T = 20 ± 2 °C, RH = 65 ± 5%), standard samples were taken from each panel to determine the appropriate physical and mechanical properties: 20 samples per shear strength test, 6 samples per MOR/MOE test, and 6 samples per dimensional changes test.

### 2.3. Physical and Mechanical Properties

Thickness, density, bending strength (MOR), and modulus of elasticity (MOE) in bending, shear strength, water absorption, and thickness swelling of plywood samples were determined according to the standards [35,36,37,38,39]. For the shear strength test, one half of the samples were tested in dry conditions and the other half in wet conditions after soaking in water at 20 ± 3 °C for 24 h. Mechanical properties of the samples, MOR and MOE in bending were carried out in parallel (‖) and perpendicular (ꓕ) directions, depending on the surface layer. Physical properties of the samples, water absorption (WA) and thickness swelling (TS), were conducted in accordance with EN-317 [39]. Before testing, the weight and thickness of each sample were measured. Conditioned samples of each type of plywood panel were fully immersed in distilled water at room temperature for 2, 24, 48, and 72 h. The samples were removed from the water, patted dry, and then measured again. The samples were weighed to the nearest 0.01 g and measured to the nearest 0.001 mm immediately.

The compression ratio (CR) of plywood panels was calculated as shown below:

(1) $$CR=(T_{V}-T_{P})/T_{V}\times 100(\%)$$

where *CR* is the compression ratio of plywood panels, *T_V_* is the total thickness of all veneers and HDPE films (mm), and *T_P_* is the thickness of the panel (mm).

Furthermore, the measurement of the core temperature that can be achieved inside the veneer package under given pressing conditions of plywood samples was undertaken. Temperature changes were measured using thermocouples connected to an PT-0102K digital multichannel device.

In addition, micromorphological properties were evaluated by microscopic imaging.

### 2.4. Statistical Analysis

Statistical analysis was conducted using SPSS software program version 22 (IBM Corp., Armonk, NY, USA). Analysis of variance (ANOVA) was performed on the data to determine significant differences at the 95% level of confidence. Duncan’s multiple range test was used to determine the significant difference between and among the groups.

## 3. Results and Discussion

### 3.1. Statistical Analysis

The influence of different factors on physical and mechanical properties of plywood was analyzed using ANOVA analysis. The results are summarized in Table 2 and Table 3. The observations made in this study and the results of the statistical analysis indicated that both mechanical and physical properties were significantly influenced by the various parameters.

### 3.2. Thickness and Density of Plywood Panels

The aim of the veneer thickness measurement was to find effects of the different conditions of pressing on the tolerance of a pressed plywood panel. In the case of using HDPE film as adhesive in the production of plywood, it is very important to choose the pressing parameters so that the thickness of finished plywood is within acceptable limits, to avoid unnecessary losses of wood raw material. The average values of the thickness, density, and moisture content of plywood samples, as well as the compression ratio, are given in Table 4.

ANOVA analysis showed that the temperature, pressure, and time of the hot pressing, as well as the type of adhesive used, significantly affects the thickness and density of the HDPE-bonded plywood panels. It can be seen that the average thickness of HDPE-bonded plywood panels made at different hot-pressing temperatures, pressures, and times is not smaller but even exceeds the thickness of control plywood using UF and PF adhesives (Table 4), which is essential for the industrial application of this technology. The smallest thickness (3.99 mm) and the highest density (619.1 kg/m^3^) had plywood samples made using PF adhesive. The largest thickness (4.70 mm) and the smallest density (536.8 kg/m^3^) had plywood samples made using HDPE film. Primarily, this can be explained by the high hot-pressing pressures of plywood samples and the increased adhesive dosage in the case of using UF and PF adhesives.

**Figure 2:**
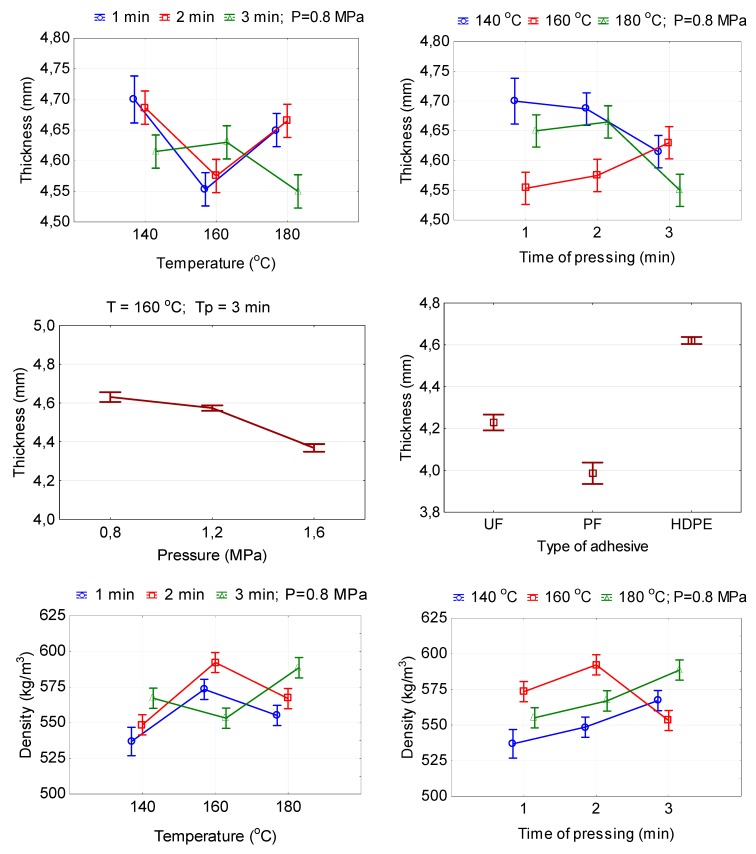

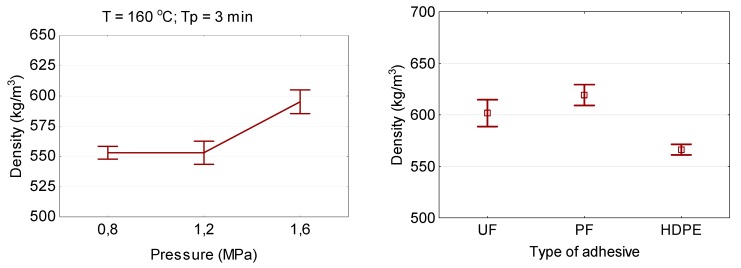
Relationship between the thickness and density of plywood and hot-pressing parameters.

As the time of the hot pressing increases from 1 to 3 min, the thickness of the plywood samples decreases at temperatures of 140 and 180 °C, and increases at a temperature of 160 °C (Figure 2). This is because at a higher hot-pressing temperature and longer time of pressing, the wood becomes more plastic and more easily compressed. The difference in thickness values of plywood samples pressed for 1 and 2 min was insignificant (*P* ≤ 0.05) based on Duncan’s test. Of course, if the thickness of the plywood samples decreases with the increase in temperature and time of pressing, then it is natural that the density of such samples, in contrast, increases (Figure 2). It was found that differences in density values of the panels pressed at temperatures of 160 and 180 °C for 2 and 3 min were insignificant (*P* ≤ 0.05) based on Duncan’s test. Nevertheless, according to the *F* values (Table 2), it can be seen that, in the ranking from highest to lowest, the hot-pressing temperature has the greatest influence on the thickness and density of the plywood samples, after that an interaction of hot-pressing temperature and hot-pressing time and finally hot-pressing time.

The hot-pressing pressure also significantly influences the thickness and density of plywood samples. With an increase in pressing pressure from 0.8 to 1.6 MPa, the thickness of the plywood samples decreases by 6.0%, and the density of samples increases by 7.6% (Figure 2). Between the pressures of 0.8 and 1.2 MPa there is no significant difference (*P* ≤ 0.05) based on Duncan’s test in the effect on the density and thickness of the plywood samples.

Nevertheless, plywood panels containing HDPE film were pressed at a lower pressure than the control panels (Table 1). In this case, the average compression ratio of plywood made using HDPE film was smaller—5.4, 3.04, and 8.05%, respectively, for 0.8, 1.2, and 1.6 MPa compared with the compression ratio of 8.5 and 13.5% for control UF and PF plywood, respectively. Moreover, plywood containing HDPE film was manufactured with 19% less adhesive spread than the adhesive spread used for the control panels. In addition, its compression ratio will be smaller because less moisture was brought with the adhesive into the veneer package, and such package, in turn, is less densified (wood is deformed more heavily).

The European Standard EN 315 [40] specifies tolerances of unsanded plywood panels for a nominal thickness of 4 mm as −0.4 mm (min) and +0.8 mm (max); i.e., the thickness of the finished unsanded plywood panels should be in the range 3.6–4.8 mm. In this study, the values of plywood thicknesses were 4.23 ± 0.04 mm and 3.99 ± 0.05 mm for panels made using UF and PF adhesives, respectively, and 4.37 (± 0.02)–4.70 (± 0.01) mm for panels made using HDPE film (Table 4), and they did not go beyond tolerances for unsanded panels in accordance with this standard.

### 3.3. Shear Strength

Average values of the dry and wet shear strength of plywood panels are presented in Table 5. The quality of the bonding of the thermoplastic adhesive and the wood surface depends on the processing details of the adhesive bonding, such as the porosity of the wood surface, the viscosity of the molten adhesive, the applied pressure, and the processing duration [41].

**Figure 3:**
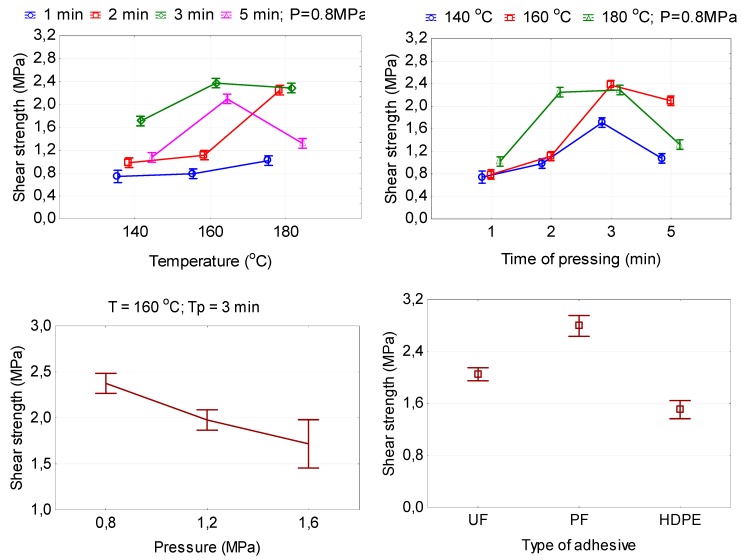
The relationship between the shear strength of plywood and hot-pressing parameters.

Figure 3 shows the effects of pressing temperature and time on the shear strength of plywood samples. The obtained strength ranged from 0.74 to 2.38 MPa, practically all of which met the European Standard EN 314-2 [38] for Class 1 (dry conditions) plywood. In this study, the bonding strength mean values obtained from the samples of HDPE-bonded plywood panels were above the limit value (1.0 MPa) indicated in the European Standard EN 314-2 [38] standard. HDPE-bonded plywood panels produced using 140 °C and 1–2 min and 160 °C and 1 min did not meet the European Standard EN 314-2 [38].

At the low temperature of 140 °C and short-term pressing time 1–2 min, the flow of HDPE is very poor; HDPE cannot permeate adequately into the vessels and cracks of veneers, resulting in worse strength. In other work [7], it was also found that lower temperatures did not promote an adequate melting of the HDPE. On the other hand, when the plywood samples were pressed at 140 °C for 3 min the result was better. de Barros Lustosa et al. [7] observed a similar trend in their study.

With increasing hot-pressing temperature from 140 to 180 °C, the shear strength of the plywood samples increased by 37.8, 127.3 and 33.9%, respectively, for pressing times of 1 min, 2 min and 3 min. This can be explained by the fact that with increasing hot-pressing temperature, more HDPE can permeate adequately into the vessels and cracks of veneers and help to enhance the bonding strength [19]. It is known that the viscosity of plastic film decreases with increasing temperatures [12,13]. This suggests that high temperatures contribute to the melting of HDPE film, which provides better fluidity, which allows for polyethylene to be more evenly distributed. In turn, this creates better conditions for the penetration of molten polyethylene into the veneer cavities and accordingly creates better conditions for the formation of mechanical locks. Accordingly, this contributes to the increase in the shear strength. Furthermore, a higher hot-pressing temperature can also decrease the proportion of hydrophilic groups in wood, thus enhancing the interfacial compatibility between hydrophilic wood and hydrophobic plastic film [25,27]. This tendency is true for the pressing time of 1–3 min. If the pressing time was increased to 5 min, then a drop in shear strength was observed with an increase in the temperature from 160 to 180 °C. Chang et al. [19] also observed that temperatures higher than 160 °C made the mechanical interlock worse and gave poorer strength to the plywood. It is obvious that at high temperature and prolonged pressing time (Figure 3), the polyethylene film is subjected to decomposition and fracturing, so that the rate of increase in the bond strength decreased.

At both temperatures, 160 and 180 °C, high strength values were obtained that exceed the minimum value according to the European Standard EN 314-2 [38]. Taking into account the energy consumed, the hot-pressing temperature of 160 °C was more economical. The highest shear strength was achieved at 160 °C when the pressing time was increased to 3 min. The smallest shear strength was achieved at 140 °C and the pressing time 1–2 min.

These results are in agreement with a study carried out by Cui et al. [11]. In this cited study, by increasing hot-pressing temperature, the bonding strength of plywood indicated a clear upward trend and then declined.

Hot-pressing time had a significant impact on the shear strength of plywood samples. Too short pressing time is insufficient for good adhesive penetration [16]. The time used should be enough for heating the inner areas of the plywood samples allowing the melting of the HDPE and the drying of the wood veneer at the same time [7]. Increasing the pressing time from 1 to 3 min leads to increased shear strength. The subsequent increase in pressing time to 5 min leads to increasing the time for production and decreasing the shear strength. This can be explained by the fact that after hot pressing for 5 min, the HDPE film was completely melted, and the thickness of the film could be reduced if the pressing time was longer than 3 min because part of the film was melted and flew out from the plywood, resulting in a lack of polyethylene film and, finally, the shear strength of plywood samples decreases. Moreover, with long-term hot pressing, the molten plastic film penetrated into the wood and, consequently, caused a decrease in the shear strength of plywood. Therefore, the hot pressing could not also be so long as to prevent thermal degradation of the wood veneer [7], decomposition and fracturing of the HDPE film. At the various hot-pressing pressures and temperatures, the highest shear strength values are observed for the pressing time of 3 min. A similar trend in the impact of pressing time on the properties of plywood is described in the work [11]. They concluded that the optimal parameters were a plastic use of 100 g/m^2^, a hot-pressing temperature of 150 °C, and a hot-pressing time of 6 min.

The pressure also played an important role because it is responsible for providing close contact between both materials and helping the flow of the HDPE into voids and irregularities of the wood veneer [7]. Therefore, high pressure is recommended for enhancement of adhesion properties with veneer [19]. In our study, with increasing pressing pressure from 0.8 to 1.6 MPa (at 160 °C and time 3 min), the shear strength decreased by 27.7%. This can be explained by the fact that with increasing the hot-pressing pressure, the more molten HDPE film was pressed into the vessels and cracks in the veneer, but less remains between the sheets of the veneer, reducing the bonding strength. Chang et al. [19] also found that when the hot-pressing pressure increased up to 1.3 MPa, the strength decreased. This phenomenon they ascribe to the fact that, with the increase in hot-pressing pressure, the ejection of HDPE resin from vessels and cracks occurs. Thus, unlike liquid adhesives such as UF and PF, the use of which requires high pressing pressure, the use of polyethylene film allows the bonding of plywood panels at significantly lower pressure (by about 50%).

The findings of our work are in good agreement with the results obtained by Smith et al. [41]. They also found that processes that kept the polypropylene molten at the surface of the wood for a longer time, higher temperature, and higher pressure achieved much better interlocking than the short process cycle.

To confirm the above-described processes of the melting and flowing of HDPE film during the hot-pressing operation, measurements of the temperature inside the veneer package for different types of adhesive used and different pressing conditions were made.

**Figure 4:**
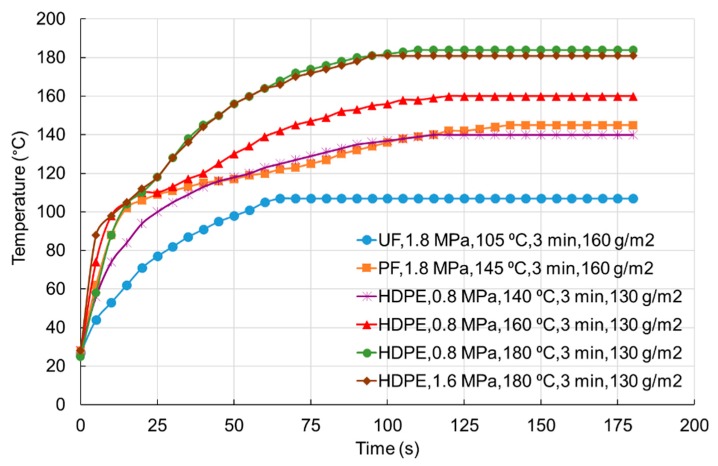
Core-layer temperature curves of three-layer plywood prepared with different adhesives at various hot-pressing conditions.

Figure 4 shows the temperature distribution inside the veneer package during the hot pressing of plywood samples for different types of adhesive used and different pressing conditions. The most slowly warmed up veneer package was the case of the manufacturing of plywood at a pressing temperature of 105 °C using UF adhesive. The temperature of 100 °C inside the veneer package is reached in 50 s from the start of pressing. The veneer package with the use of PF adhesive in which pressing is carried out already at a higher temperature of 145 °C warmed up faster. The temperature of 100 °C inside the veneer package was reached in 14 s, which is almost 3.5 times faster than in the case of pressing UF plywood. The main factor determining the speed of heating the veneer package with HDPE film is the pressing temperature. The melting temperature of the HDPE film is 135 °C. Such temperature is achieved inside the veneer package in 90, 56, and 34 s at pressing temperatures of 140, 160, and 180 °C, respectively. With the increase in the pressing temperature, the heating rate of the veneer package increases. This has an effect on the bonding strength of plywood using HDPE film. From Figure 3 and Table 5, it can be seen that the temperature of 140 °C and the pressing time of 1 and 2 min are insufficient to warm the package, to melt the plastic film, and to allow it to flow over the surface of the veneer sheet. At a temperature of 140 °C, the film’s fluidity is insufficient to move inside the pores of the wood and to form mechanical adhesive bonds, which adversely affects the bonding strength (Figure 3, Table 5). For temperatures of 160 and 180 °C, the pressing time of 1 min is also undesirable because the bonding strength is practically equal to the permissible value of 1.0 MPa in accordance with European Standard EN 314-2 [38].

**Figure 5:**
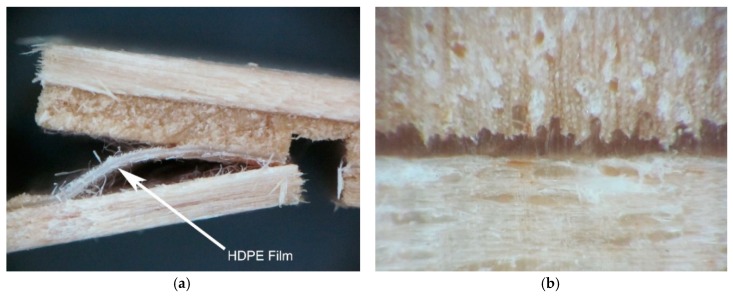
Micrograph of the HDPE-bonded plywood samples (**a**) prepared at 140 °C and 1 min after shear strength test; (**b**) prepared at 160 °C and 3 min.

Figure 5 shows microscopic images of alder veneer bonded with HDPE film. In the plywood samples prepared at 140 °C and a pressing time of 1 min, a bondline was observed between the adjacent two veneers, indicating that the penetration of HDPE films into the veneer surfaces was not desirable, which could easily cause the delamination of HDPE films from the veneer surfaces (Figure 5a). This image clearly indicates the lack of chemical bonds between the plastic film and wood substance because the HDPE film could be easily separated mechanically from the wood after a short immersion in water. This was an indication of poor adhesion of the HDPE film to the wood surface. The lack of chemical bonds between the plastic films and the surface of wood are also indicated by other authors [13,15,19,32,41]. Therefore, the plywood samples prepared using 140 °C and a pressing time of 1 min had the lowest shear strength in Figure 3 and Table 5.

In the plywood samples prepared using higher temperatures of 160 and 180 °C and a longer pressing time of 2–3 min, the penetration of HDPE films into the veneer surfaces was better (Figure 5b) than that in the samples prepared using 140 °C and a pressing time of 1 min. It can be seen that HDPE film flowed and penetrated into the vessels of wood veneer during the hot-pressing stage, forming a continuous bondline and mechanical interlock structure (Figure 5b). The better penetration produces stronger bonded joints in the samples. Therefore, the plywood samples prepared at 160 °C and a pressing time of 3 min had the highest shear strength in Table 5.

In another study [32], it was also shown that the polypropylene melted during hot pressing and made good contact with veneer surfaces penetrating into the lumina of wood cells, lathe checks, and other spaces open on the veneer surface. These authors indicated that the anchoring effect of polypropylene, which had penetrated into various wood elements and spaces in the veneer, contributed dominantly to the gluability [32]. Therefore, because wood is a porous material, a mechanical interlock is the most likely bonding mechanism involved [13,15,19,32,41].

### 3.4. Bending Strength and Modulus of Elasticity in Bending

The average values of the bending strength and modulus of elasticity (MOE) in the bending of plywood samples are presented in Table 6.

**Figure 6:**
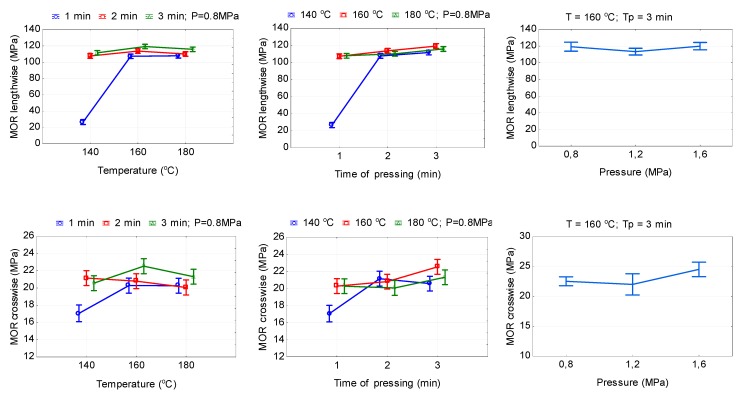
The relationship between bending strength and hot-pressing parameters (lengthwise = in parallel and crosswise = in perpendicular directions).

The temperature, pressure, and time of hot pressing significantly (*P* ≤ 0.05) affect the bending strength in both parallel MOR (‖) and perpendicular MOR (ꓕ) directions of HDPE-bonded plywood samples (Table 2). With an increase in the pressing temperature from 140 to 180 °C and pressing time from 1 to 3 min, MOR (‖) and MOR (ꓕ) increased by 304.1 and 3.8% and 14.9 and 0.5%, respectively (Figure 6). For a pressing time of 2 min with an increase in the pressing temperature from 140 to 180 °C, MOR (‖) increased and MOR (ꓕ) decreased by 2.3 and 9.4%, respectively. As can be seen from Table 6, MOR (‖) values exceed the MOR (ꓕ) values by a factor of more than five, except the pressing conditions of 140 °C and 1 min. A similar trend can also be observed in the case of increasing the pressing time from 1 to 3 min (Figure 6). In this case, the MOR (‖) and MOR (ꓕ) increased by 318.0, 11.2, and 7.3% and 18.9, 11.9, and 4.0%, respectively, for pressing temperatures of 140, 160 and 180 °C. The differences in the values of MOR (‖) and MOR (ꓕ) for the temperatures of 160 and 180 °C are insignificant. Therefore, from an economic point of view, it is more expedient to hot-press at a temperature of 160 °C. The smallest value of MOR (‖) was obtained at a hot-press pressure of 1.2 MPa (113.4 MPa), and the highest values of MOR (‖) were obtained at 0.8 MPa (119.3 MPa) and 1.6 MPa (119.9 MPa) (Table 6). The difference in the values of MOR (‖) for the pressures of 0.8 and 1.6 MPa is insignificant. With increasing pressing pressure from 0.8 to 1.6 MPa, the value of MOR (ꓕ) increased by 8.9%. The difference in the values of MOR (ꓕ) for the pressures of 0.8 and 1.2 MPa is insignificant.

**Figure 7:**
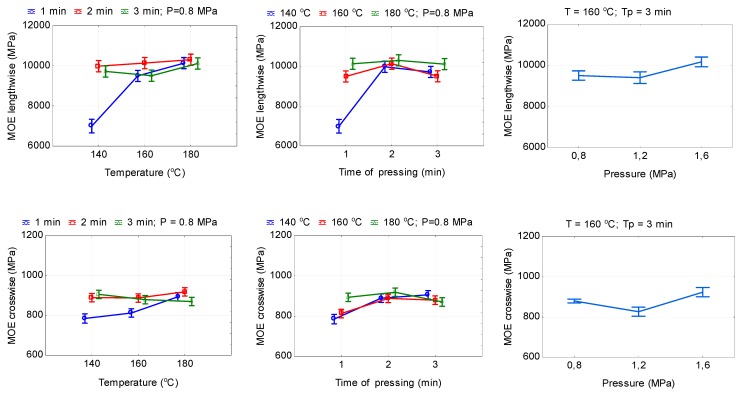
The relationship between the modulus of elasticity in bending and hot-pressing parameters (lengthwise = in parallel and crosswise = in perpendicular directions).

The temperature, pressure, and time of hot pressing also significantly (*P* ≤ 0.05) affect the MOE in bending in both parallel MOE (‖) and perpendicular MOE (ꓕ) directions of HDPE-bonded plywood samples (Table 2). With an increase in the pressing temperature from 140 to 180 °C and pressing time from 1 to 3 min, the values of MOE (‖) and MOE (ꓕ) increased by 44.9, 3.2, and 4.1% and 12.4, 1.9, and 4.8% (reduction), respectively (Figure 7). With increasing the pressing time from 1 to 2 min, MOE (‖) values initially increased, and further increase in the pressing time to 3 min led to a decrease in MOE (‖) (Figure 7). The values of MOE (ꓕ) increased by 6.2–7.8% with an increase in the pressing time from 1 to 3 min. In addition, the difference in the values of MOR (ꓕ) for the pressing times of 2 and 3 min is insignificant.

With increasing hot-pressing pressure from 0.8 to 1.6 MPa, the values of MOE (‖) increased by 7%. The difference in the values of MOE (‖) for the pressing pressures 0.8 and 1.2 MPa is insignificant. The lowest values of MOE (ꓕ) were observed at a pressing pressure of 1.2 MPa (826.4 MPa), and the highest at a pressure of 1.6 MPa (923.8 MPa) (Table 6).

**Figure 8:**
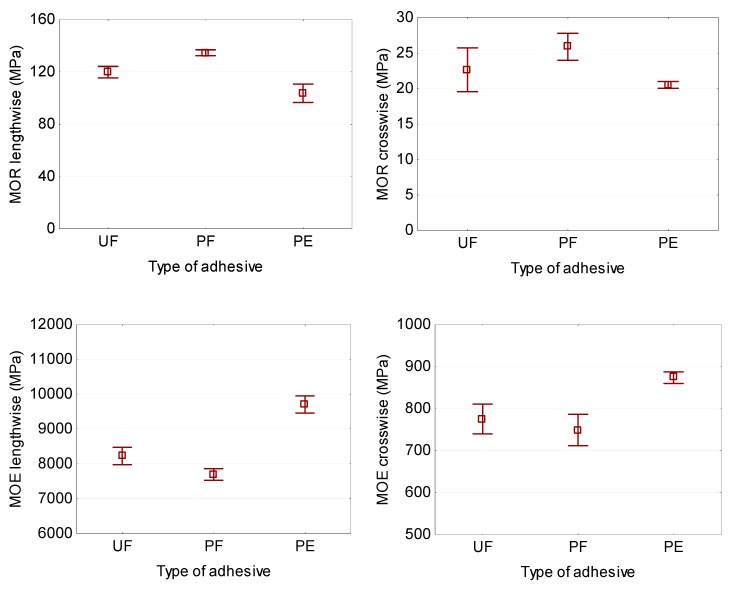
Bending strength and modulus of elasticity in the bending of plywood panels bonded with different types of adhesive (lengthwise = in parallel and crosswise = in perpendicular directions).

It has been established that the type of adhesive also significantly (*P* ≤ 0.05) affects the mechanical properties of plywood samples, MOR (‖) and MOR (ꓕ), MOE (‖) and MOE (ꓕ) (Table 2). The highest MOR (‖) and MOR (ꓕ) values were obtained for PF adhesive (134.6 and 25.9 MPa, respectively), followed by UF adhesive (119.8 and 22.7 MPa, respectively) and HDPE film (103.6 and 20.5 MPa, respectively) (Table 6, Figure 8). The lowest MOE (‖) and MOE (ꓕ) values were recorded for PF adhesive (7698.9 and 748.6 MPa, respectively) and UF adhesive (8228.2 and 775.2 MPa, respectively), and the highest for HDPE films (9702.9 and 873.6 MPa, respectively) (Table 6, Figure 8). The differences in the values of MOE (‖) and MOE (ꓕ) for the UF and PF adhesives are insignificant.

The effect of adhesive could be explained by the quite different properties between thermoplastic (HDPE) and thermoset (UF and PF) polymers. HDPE is a well-known thermoplastic material, because of the inherent molecular structure the thermoplastic polymers are more plastic and show higher toughness than thermosets [20].

Another study stated that the average mechanical properties of polystyrene-bonded plywood panels tend to increase when the pressing time and temperature are increased during production [16].

Mean values obtained for bending strength and MOE of plywood panels were higher than the limit values for structural purpose solid wood panels (32 MPa for MOR (‖) and 5 MPa for MOR (ꓕ), 9000 MPa for MOE (‖) and 600 MPa for MOE (ꓕ)) indicated in European Standard EN 13353 [42] for panels having thicknesses up to 20 mm.

The bending strength and MOE values of HDPE-bonded plywood panels were (Table 6):

- 26.7–119.9 MPa and 6996.2–10,307.7 MPa, respectively, when parallel to the grain direction;
- 17.5–24.5 MPa and 796.8–923.8 MPa, respectively, when perpendicular to the grain direction.

**Figure 9:**
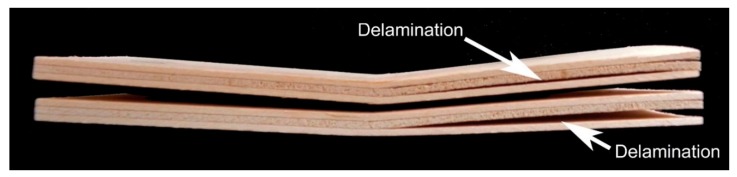
Delamination in the plywood samples prepared at 140 °C and 1 min during the determination of bending strength.

In all specimens, the determined bending strength and MOE values perpendicular to the grain direction were higher than the lower limiting value of 5 and 600 MPa, respectively. The plywood panels produced at 140 °C and 1 min had the smallest bending strength (MOR (‖) and MOE (‖)) values in Table 6 and did not meet the European Standard EN 13353 [42]. These panels had the least maximum load to failure, 188.9 N on average, and the least deflection to failure at 1.92 mm. As can be seen from Figure 9, the generated delamination crack longitudinally extended through the sample and this significantly reduced the maximum failure load. This can diminish the bending strength of plywood. As was shown above, the shear strength values of plywood samples bonded at the hot-pressing temperature of 140 °C and pressing time of 1 min were also the smallest.

### 3.5. Thickness Swelling and Water Absorption

The average values of TS and WA of plywood samples are given in Table 7.

The pressing temperature and time insignificantly (*P* ≤ 0.05) affect the TS of the HDPE-bonded plywood samples (Table 3). The smallest TS was observed at a pressing time of 2 min, and the difference in the values of TS for the pressing times of 1 and 3 min was insignificant. With increasing time of soaking, the TS increases (Table 7). The smallest TS was observed during soaking for 2 h, and then the TS is increased by 42.7–46.5%. The differences in the values of TS for the time of soaking 24, 48, and 72 h were insignificant.

The pressing pressure significantly (*P* ≤ 0.05) affects the TS of HDPE-bonded plywood samples (Table 3). With increasing pressing pressure from 0.8 to 1.6 MPa, the values of TS increased by 17.7%. The difference in the values of TS for the pressing pressures 0.8 and 1.2 MPa was insignificant.

The type of adhesive had a significant (*P* ≤ 0.05) effect on TS (Table 3). The smallest value of TS was observed in plywood samples using HDPE film (8.11%), the higher value in samples using UF adhesive (8.70%) and the highest value in samples using PF adhesive (11.09%) (Table 7). This was attributed to the hydrophobic character of the plastic film. This result was also associated with the hot-pressing condition. A higher hot-pressing temperature for wood-based composites can reduce hydrophilic groups in raw materials, which contributes to reducing the water absorption and the TS of plywood panels [25,27]. In this research, the hot-pressing temperature of HDPE-bonded plywood samples was higher than PF-bonded samples, while the hot-pressing temperature of PF-bonded samples was higher than that of UF-bonded samples.

The temperature and time of hot pressing significantly affect the WA of HDPE-bonded plywood samples (Table 3). The lowest values of WA were found at 160 °C (56.03%), and higher values were obtained at temperatures of 140 and 180 °C (58.5% and 57.3%, respectively) (Table 7). The difference in the values of WA for the pressing temperatures of 140 and 180 °C was insignificant. The smaller value of WA was observed at the pressing time of 3 min (56.5%), and higher value at a pressing time of 1 min (58.3%). The difference in the values of WA for the pressing time of 2 and 3 min was insignificant. With increasing time of soaking in water from 2 to 72 h, the WA gradually increased by 68.3%. The largest increase in WA occurs during the first 2 h of soaking in water.

The pressing pressure affects WA insignificantly (Table 3). The type of adhesive had a significant effect on water absorption. The smallest WA was observed in plywood samples using UF adhesive (54.3%), intermediate in samples using HDPE film (57.1%), and the highest in samples using PF adhesive (59.7%) (Table 7).

### 3.6. Effect of Adhesive Types on the Physical and Mechanical Properties of Plywood

Plywood bonded with UF and PF resins were manufactured for comparison with plywood bonded with HDPE film. All plywood contained approximately equal adhesive dosage. Table 8 summarizes the physical and mechanical properties of the three types of plywood.

The mechanical properties of HDPE-, UF-, and PF-bonded plywood panels were comparable despite the fact that these three polymers have considerably different mechanical properties, which affect the final properties of the panels. The lower TS and WA of the plywood could be explained by the fact that HDPE film filled the micropores of the wood veneers and covered a larger surface area of the hygroscopic wood component and thus prevents water penetration into the wood veneers [12,13].

HDPE-bonded plywood samples were made using a lower adhesive dosage (130 g/m^2^) and at a lower hot-pressing pressure than UF and PF plywood samples. However, their properties are not inferior to these traditional plywood. In addition, HDPE-bonded plywood can be attributed to environmentally-friendly plywood. The formaldehyde emission of the plywood made from recycled plastics is very low; compared with that of ordinary plywood made with urea–formaldehyde resin, the amount of emission is almost zero [11]. In addition, the use of HDPE film makes plywood more flexible and simplifies the production of different bent constructions from plywood [21].

The values of the mechanical properties of the plywood panels obtained in this work were significantly higher than those obtained in other works [13,16,18,19,24], which mainly used poplar and very rarely eucalyptus [25], as well as different films. It can be assumed that wood species may have an effect on the ability to be bonded with a thermoplastic film.

## 4. Conclusions

The thermoplastic polymers were successfully used for the bonding of alder plywood. The findings of this work indicate that HDPE film adhesive gave bending strength, MOE in bending, and shear strength values of alder plywood panels that are comparable to those obtained with traditional UF and PF adhesives. Moreover, this is despite the fact that the adhesive dosage and pressing pressure were less than when UF and PF adhesives were used. Among the HDPE-bonded alder plywood panels, all the highest mechanical properties values were obtained for panels produced with high pressing temperature and pressing time. The highest shear strength was achieved at 160 °C when the pressing time was increased to 3 min. The smallest shear strength was achieved at 140 °C and a pressing time of 1–2 min. Environmentally-friendly high-density polyethylene-bonded formaldehyde-free alder plywood panels have been successfully produced using thermoplastic polymers as an adhesive.

## Figures and Tables

**Table 1:** Manufacturing conditions of plywood.

Test No.	Manufacturing Conditions				
	Adhesive Type	Adhesive Spread Rate (g/m^2^)	Pressing Pressure (MPa)	Pressing Temperature (°C)	Pressing Time (min)
1	HDPE	130	0.8	140, 160, 180	1, 2, 3, 5
2	HDPE	130	0.8, 1.2, 1.6	160	3
4	UF	160	1.8	105	3
5	PF	160	1.8	145	3

**Table 2:** ANOVAs of the variable parameters on the mechanical and physical properties of plywood panels.

Source	Thickness		Density		MOR (‖)		MOR (ꓕ)		MOE (‖)		MOE (ꓕ)		Shear Strength	
	*F*	*P*	*F*	*P*	*F*	*P*	*F*	*P*	*F*	*P*	*F*	*P*	*F*	*P*
*T*	23.419	0.000 *	29.001	0.000 *	470.672	0.000 *	10.349	0.000 *	60.390	0.000 *	10.726	0.000 *	196.558	0.000 *
*Tp*	8.907	0.001 *	13.653	0.000 *	544.381	0.000 *	20.852	0.000 *	60.400	0.000 *	34.705	0.000 *	411.222	0.000 *
*T* × *Tp*	15.852	0.000 *	28.080	0.000 *	311.957	0.000 *	7.087	0.000 *	34.411	0.000 *	11.866	0.000 *	98.263	0.000 *
*P*	314.877	0.000 *	54.816	0.000 *	3.973	0.041 *	7.858	0.007 *	18.195	0.000 *	50.216	0.000 *	5.665	0.007 *
*A*	417.616	0.000 *	33.237	0.000 *	5.540	0.006 *	25.482	0.000 *	22.496	0.000 *	27.623	0.000 *	22.974	0.000 *

Note: T = temperature of pressing; Tp = time of pressing; P = pressure of pressing; A = type of adhesive; *F* = *F* value; MOR (‖) = bending strength in parallel direction; MOR (ꓕ) = bending strength in perpendicular direction; MOE (‖) = modulus of elasticity in bending in parallel direction; MOE (ꓕ) = modulus of elasticity in bending in perpendicular direction. * Significant difference at the 5% level (*P* ≤ 0.05).

**Table 3:** ANOVAs of the variable parameters on the physical properties of plywood panels.

Source	TS (2 h)		TS (24 h)		TS (48 h)		TS (72 h)		WA (2 h)		WA (24 h)		WA (48 h)		WA (72 h)	
	*F*	*P*	*F*	*P*	*F*	*P*	*F*	*P*	*F*	*P*	*F*	*P*	*F*	*P*	*F*	*P*
*T*	1.256	0.296 ^ns^	1.378	0.264 ^ns^	1.268	0.292 ^ns^	1.580	0.219 ^ns^	2.049	0.142 ^ns^	2.691	0.080 ^ns^	0.631	0.537 ^ns^	8.609	0.001 *
*Tp*	3.023	0.060 ^ns^	2.065	0.140 ^ns^	0.436	0.650 ^ns^	0.221	0.802 ^ns^	1.298	0.284 ^ns^	3.459	0.041 *	1.426	0.252 ^ns^	3.955	0.027 *
*T* × *Tp*	2.627	0.063 ^ns^	1.230	0.311 ^ns^	2.542	0.070 ^ns^	1.258	0.302 ^ns^	1.421	0.251 ^ns^	2.392	0.083 ^ns^	0.971	0.416 ^ns^	2.927	0.045 *
*P*	3.649	0.051 ^ns^	10.007	0.002 *	14.203	0.000 *	14.974	0.000 *	0.389	0.684 ^ns^	0.978	0.399 ^ns^	2.670	0.102 ^ns^	9.711	0.002 *
*A*	15.427	0.000 *	75.756	0.000 *	60.828	0.000 *	76.271	0.000 *	7.921	0.001 *	19.885	0.000 *	13.428	0.000 *	3.561	0.035 *

Note: T = temperature of pressing; Tp = time of pressing; P = pressure of pressing; A = type of adhesive; *F* = *F* value. * Significant difference at the 5% level (*P* ≤ 0.05); ^ns^ = not significant.

**Table 4:** Thickness and density of plywood panels.

Type of Adhesive	Pressing Conditions			Moisture Content (%)	Compression Ratio CR (%)	Thickness (mm)	Density (kg/m^3^)
	Pressure (MPa)	Temperature (°C)	Time (min)				
UF	1.8	105	3	5.0	8.5	4.23 (0.04) *	601.7 (12.42)
PF	1.8	145	3	3.5	13.5	3.99 (0.05)	619.1 (9.67)
HDPE	0.8	140	1	4.1	4.2	4.70 (0.01)	536.8 (23.05)
			2	3.8	4.8	4.69 (0.04)	548.5 (7.79)
			3	3.7	5.2	4.62 (0.04)	567.2 (8.02)
		160	1	3.8	5.3	4.55 (0.06)	573.4 (9.04)
			2	3.5	7.1	4.58 (0.01)	592.2 (4.09)
			3	3.1	6.1	4.63 (0.02)	553.2 (5.02)
		180	1	3.4	4.5	4.65 (0.02)	555.1 (5.73)
			2	3.2	5.7	4.67 (0.01)	567.0 (10.34)
			3	3.2	5.7	4.55 (0.03)	588.6 (4.48)
	1.2	160	3	2.8	3.0	4.57 (0.01)	553.2 (9.06)
	1.6	160	3	2.9	8.1	4.37 (0.02)	595.2 (9.32)

* Values in parenthesis are standard deviations. UF: urea–formaldehyde. PF: phenol–formaldehyde.

**Table 5:** Shear strength values of plywood panels pressed using different types of adhesive and pressing conditions.

Type of Adhesive	Pressing Conditions			Shear Strength (MPa)	
	Pressure (MPa)	Temperature (°C)	Time (min)	Dry Test	Wet Test
UF	1.8	105	3	2.99 (0.15) *	2.05 (0.14)
PF	1.8	145	3	2.95 (0.10)	2.79 (0.23)
HDPE	0.8	140	1	1.72 (0.14)	0.74 (0.12)
			2	1.73 (0.14)	0.99 (0.07)
			3	2.44 (0.09)	1.71 (0.12)
			5	2.01 (0.13)	1.08 (0.05)
		160	1	1.60 (0.11)	0.79 (0.05)
			2	1.94 (0.09)	1.03 (0.10)
			3	2.99 (0.12)	2.38 (0.15)
			5	2.87 (0.11)	2.1 (0.14)
		180	1	1.73 (0.18)	1.02 (0.08)
			2	2.64 (0.08)	2.25 (0.10)
			3	2.67 (0.16)	2.29 (0.12)
			5	2.38 (0.23)	1.32 (0.11)
	1.2	160	3	2.91 (0.08)	1.97 (0.13)
	1.6	160	3	2.74 (0.14)	1.72 (0.56)

* Values in parenthesis are standard deviations.

**Table 6:** MOR and MOE values of plywood panels pressed using different types of adhesive and pressing conditions.

Type of Adhesive	Pressing Conditions			MOR (MPa)		MOE (MPa)	
	Pressure (MPa)	Temperature (°C)	Time (min)	MOR (‖)	MOR (ꓕ)	MOE (‖)	MOE (ꓕ)
UF	1.8	105	3	119.8 (4.2) *	22.7 (2.5)	8228.2 (232.2)	775.2 (28.4)
PF	1.8	145	3	134.6 (2.2)	25.9 (1.5)	7698.9 (160.8)	748.6 (30.1)
HDPE	0.8	140	1	26.7 (4.3)	17.5 (0.4)	6996.2 (886.0)	796.8 (37.8)
			2	107.6 (2.8)	21.3 (0.7)	9986.6 (191.5)	891.8 (14.0)
			3	111.6 (2.2)	20.8 (0.2)	9722.7 (150.4)	911.7 (28.3)
		160	1	107.3 (2.6)	20.1 (0.5)	9501.5 (309.9)	816.2 (17.1)
			2	113.7 (3.4)	20.8 (0.2)	10,141.8 (170.8)	891.7 (16.3)
			3	119.3 (5.1)	22.5 (0.6)	9511.0 (214.6)	879.7 (7.3)
		180	1	107.9 (3.3)	20.1 (0.8)	10,136.9 (278.1)	895.7 (17.6)
			2	110.1 (3.1)	19.3 (1.3)	10,307.7 (329.6)	908.4 (37.5)
			3	115.8 (2.6)	20.9 (0.2)	10,119.9 (322.2)	867.9 (15.0)
	1.2	160	3	113.4 (3.9)	22.0 (1.4)	9407.4 (272.2)	826.4 (18.2)
	1.6	160	3	119.9 (4.2)	24.5 (1.0)	10,173.7 (225.5)	923.8 (18.1)

* Values in parenthesis are standard deviations.

**Table 7:** Thickness swelling (TS) and water absorption (WA) of plywood panels pressed using different types of adhesive and pressing conditions.

Type of Adhesive	Pressing Conditions			Physical Properties							
	P (MPa)	T (°C)	Time (min)	TS (2 h)	TS (24 h)	TS (48 h)	TS (72 h)	WA (2 h)	WA (24 h)	WA (48 h)	WA (72 h)
UF	1.8	105	3	6.03	9.38	9.70	9.70	35.84	53.12	60.19	67.94
PF	1.8	145	3	7.94	11.84	12.21	12.38	37.29	61.29	67.67	72.66
HDPE	0.8	160	3	6.15	8.82	8.82	9.00	38.86	51.38	59.51	70.48
	1.2			5.90	9.22	9.37	9.37	39.08	52.75	61.51	71.76
	1.6			7.02	10.19	10.61	10.80	40.70	51.83	59.32	66.75
	0.8	140	1	–	–	–	–	–	–	–	–
			2	5.97	8.29	8.54	8.64	44.46	56.14	63.88	74.30
			3	6.53	8.63	8.78	8.85	42.43	53.62	61.22	71.95
	0.8	160	1	6.51	8.90	9.55	9.33	44.58	56.20	63.41	70.71
			2	5.06	8.52	8.82	8.89	38.85	52.10	58.87	67.35
			3	6.15	8.82	8.82	9.00	38.86	51.38	59.51	70.48
	0.8	180	1	5.95	8.35	8.49	8.46	42.52	54.11	61.10	73.60
			2	6.18	8.65	8.86	8.86	42.33	53.51	61.70	71.23
			3	6.08	8.97	9.08	8.94	43.59	54.26	61.50	68.74

– the samples delaminated.

**Table 8:** Physical and mechanical properties of plywood panels bonded with different adhesives *.

Adhesive Type	T (mm)	D (kg/m^3^)	Dry SS (MPa)	Wet SS (MPa)	MOR (‖) (MPa)	MOR (ꓕ) (MPa)	MOE (‖) (MPa)	MOE (ꓕ) (MPa)	TS (24 h) (%)	WA (24 h) (%)
UF	4.23	601.7	2.99	2.05	119.8	22.7	8228.2	775.2	9.38	53.12
PF	3.99	619.1	2.95	2.79	134.6	25.9	7698.9	748.6	11.84	61.29
HDPE **	4.63	553.2	2.99	2.38	119.3	22.5	9511.0	879.7	8.82	51.38

* T = thickness; D = density; SS = shear strength; MOR (‖) = bending strength in parallel direction; MOR (ꓕ) = bending strength in perpendicular direction; MOE (‖) = modulus of elasticity in bending in parallel direction; MOE (ꓕ) = modulus of elasticity in bending in perpendicular direction; TS = thickness swelling; WA = water absorption. ** The properties values for HDPE film are indicated for HDPE-bonded samples at 160 °C and 3 min.
